# Radioresistance of Brain Tumors

**DOI:** 10.3390/cancers8040042

**Published:** 2016-03-30

**Authors:** Kevin Kelley, Jonathan Knisely, Marc Symons, Rosamaria Ruggieri

**Affiliations:** 1 Radiation Medicine Department, Hofstra Northwell School of Medicine, Northwell Health, Manhasset, NY 11030, USA; kkelley1@northwell.edu (K.K.); jknisely@northwell.edu (J.K.); 2 The Feinstein Institute for Molecular Medicine, Hofstra Northwell School of Medicine, Northwell Health, Manhasset, NY 11030, USA

**Keywords:** radiation therapy, radioresistance, brain tumors

## Abstract

Radiation therapy (RT) is frequently used as part of the standard of care treatment of the majority of brain tumors. The efficacy of RT is limited by radioresistance and by normal tissue radiation tolerance. This is highlighted in pediatric brain tumors where the use of radiation is limited by the excessive toxicity to the developing brain. For these reasons, radiosensitization of tumor cells would be beneficial. In this review, we focus on radioresistance mechanisms intrinsic to tumor cells. We also evaluate existing approaches to induce radiosensitization and explore future avenues of investigation.

## 1. Introduction

### 1.1. Radiotherapy and Radioresistance of Brain Tumors

Radiation therapy is a mainstay in the treatment of the majority of primary tumors of the central nervous system (CNS). However, the efficacy of this therapeutic approach is significantly limited by resistance to tumor cell killing after exposure to ionizing radiation. This phenomenon, termed radioresistance, can be mediated by factors intrinsic to the cell or by the microenvironment. One approach to overcome radioresistance has been to alter the parameters under which radiotherapy is delivered and another has centered on understanding the molecular complexity that underlies radioresistance with the aim of developing targeted therapies. This review summarizes these efforts in the context of primary brain tumors.

### 1.2. Effects of Radiation on Normal Brain Tissue

The inherent radiosensitivity of normal brain tissue manifests as late toxicity in the form of radionecrosis and is evident in some patients after completing CNS-directed radiotherapy. This is not to be confused with the term “pseudoprogression” which describes a radiological finding thought to represent radiation damage to the tumor itself rather than normal brain toxicity [1]. In contrast to pseudoprogression, radionecrosis does not resolve spontaneously and is often associated with outward clinical findings, such as recurrent seizure and focal neurological symptoms. On histological review, gliosis, apoptotic endothelial cell death and white and gray matter necrosis are evident. Additionally, there are T2-signal abnormalities, indicating increased vascular permeability that lead to white matter edema that is visible on MRI [2,3]. One of the key mechanisms thought to contribute to radionecrosis is radiation-triggered endothelial cell and oligodendrocyte demise primarily through apoptosis. This leads to demyelination and microenvironmental dysfunction through chronic hypoxia and inefficient vascular remodeling. This process contributes to the perpetuation of chronic inflammatory mediators such as tumor necrosis factor-α and increased transactivation by NF-κB [2,4,5,6,7]. Post-irradiation vascular insufficiency leads to chronic ischemia, which contributes to worsening hypoxia, nutrient deficiency, increased oxidative stress and reactive oxygen species (ROS) generation, toxic to normal brain parenchyma in the vicinity. As reactive oxygen species are central to damage caused by radiation [8], antioxidants have been investigated for their potential protective effect of normal cells in the context of other malignancies. However, results of clinical trials using antioxidants have been disappointing and new opportunities are being evaluated, such as agents affecting pathways leading to bystander genome destabilization [9].

## 2. Dose Escalation and Altered Fractionation to Combat Radioresistance

Modulation of radiation’s delivery is an approach that can be readily pursued through alteration of total cumulative dose delivered or adjustment of the fractionation schedule in order to exploit differences between the radiobiology of the tumor and normal tissues. Data-driven incremental dose escalation has improved local control for many different tumor types, including prostate and lung carcinomas [10,11,12,13,14,15], but dose escalation for primary brain tumors has been an exercise in futility [16].Technical advances in radiation delivery [intensity modulated radiation therapy (IMRT) and protons] that limit the dose to peritumoral brain has permitted hope to exist that this approach may still have merit [17]. Mathematical modeling and simulation of glioblastoma radiobiology have identified ostensibly more efficacious radiation fractionation schedules compared to standard fractionation [18,19,20,21]. However, clinical trials evaluating non-standard radiotherapy fractionation schedules for glioblastoma have not shown promise [22,23,24,25,26].

Computerized approaches to image registration, both at the time of radiotherapy planning and at the time of radiation delivery, have permitted more accurate target volume definition and more accurate treatment delivery [27,28]. Because in the CNS there are specialized structures with more limited radiation tolerance [29,30,31,32,33], these technical advances have allowed for smaller planning target volume margins to cause less normal tissue dose exposure [34,35,36,37]. Computerized control of radiation delivery using approaches, such as IMRT and volumetric modulated arc therapy, have permitted increasingly complex treatments that reduce the dose absorbed to these critical normal tissues, while delivering a tumoricidal dose to the target in acceptably brief treatment appointments [38,39,40,41].

In recent years, stereotactic radiosurgery (SRS) has been evolving to safely deliver ablative high doses of focused radiation with high precision to the tumor or post-operative tumor bed, while limiting dose to critical adjacent normal structures. This modality has been tested and found to be useless, and possibly harmful in the primary management of glioblastoma [41,42,43]. Inadequate data exist to assess its value in the management of inherently radioresistant (exposed to prior RT) malignant glioma recurrences [42,44,45], but it appears to be useful for localized residual foci and recurrences of tumors such as ependymomas and medulloblastoma [46]. SRS has become a popular treatment strategy for non-infiltrative CNS tumors as well as brain metastases [47,48]. In the latter case, the toxic side effects of whole brain radiation, which includes profound cognitive impairment, can be avoided by treating patients with SRS, while not sacrificing local tumor control [49,50]. Nevertheless, for most primary CNS neoplasms, conventionally fractionated radiotherapy remains the current standard of care [51].

## 3. Rationale for Pharmaceutical Radiosensitizers

An alternative strategy to combat brain tumor resistance to radiation therapy is through the administration of tumor-specific radiosensitizing agents prior to or during radiation treatment in order to enhance the sensitivity of the tumor target, while not affecting the intrinsic tolerance of in- or near-field normal brain. A framework to characterize and understand potential interactions between traditional chemotherapeutic agents and therapeutic ionizing radiation was described by Steel and colleagues in 1979 [52,53]. The concept of synergy or supra-additivity emerged, as illustrated by the classic isobologram described by Steel and Peckham [54].

In the management of pediatric brain tumors, radiosensitization is desirable to accomplish dose reduction, given the inherent adverse sensitivity of the developing brain, spine and encasing osseous structures to ionizing radiation. Processes critical to neuronal development, cognition, Intelligence Quotient (IQ), and achievement of developmental milestones can be severely impacted. Although the prognosis of patients with medulloblastoma, the most common pediatric brain tumor, is in general very good, with a 5 years tumor free survival of 75% to more than 95% in some subtypes [55], this population suffers dramatically from the deleterious effects of radiation on the normal developing brain. Overall normal development and growth are negatively impacted with the craniospinal irradiation used for this disease. Decreased IQ and stunted skeletal growth and deformities, along with alopecia and hormonal deficits, severely compromise the child’s quality of life far into adulthood [56]. A tumor-specific radiosensitizer would be useful in this case to enable reduction of the craniospinal dose, while still maintaining the high probability of cure currently afforded with modern therapy. Although adverse effects on growth and development are not an issue in adult populations, targeted radiosensitization is also desirable when treating adult patients with malignant CNS tumors to minimize the risk of radionecrosis or other dose-related adverse side effects.

An example of a radiosensitizer currently used with concurrent radiotherapy as a standard of care in the treatment of glioblastoma is temozolomide (TMZ). Through pioneering work conducted in the 1970s–1980s in the UK, the compound CCRG81045 gradually found its way into early phase I and II clinical trials, where it was shown to be safe and effective in enhancing clinical and radiologic responses in small groups of patients with GBM [57,58]. EORTC 26981, known colloquially as the “Stupp trial”, represented the first phase III randomized trial where 573 patients with newly diagnosed GBM, after maximal safe surgical resection, were treated concurrently with adjuvant radiation therapy to 60 Gy and daily TMZ to a dose of 75 mg/m^2^, followed by 6 cycles of adjuvant monthly TMZ at a dose of 150–200 mg/m^2^ for 5 consecutive days per cycle vs. adjuvant RT alone. Median and 5 year-overall survival favored the combined TMZ + radiation arm at 14.6 months *vs*. 12.1 months and 9.8 *vs*. 1.9%, respectively [59]. The mechanism of action through which TMZ radiosensitizes glioma cells is through the DNA damaging effects of alkylation at O-6 of the guanine residue. Tumor cells can remove and repair this type of damage through expression of O(6)-methylguanine-DNA methyltransferase (MGMT), thus escaping cell death. However, hypermethylation of the MGMT promotor epigenetically silences the gene leaving cells sensitized to the damaging effects of alkylating chemotherapy [60]. Screening for MGMT promoter methylation has been shown to be prognostic for improved survival in response to therapy [60,61,62].

Identifying effective radiosensitizers requires an understanding of the underlying molecular causes of radioresistance. Much progress has been made in this area and the remainder of this review will summarize our current knowledge of the molecular causes of radioresistance.

## 4. Tumor Heterogeneity

Radioresistance is caused by a variety of factors that include the intrinsic biology that arises from the aberrant genetic makeup of tumor cells, the extensive heterogeneity of brain tumors [63,64] and the tumor microenvironment, which fosters and supports conditions that limit the response to radiation. Tumors contain heterogeneous populations of stromal cells that support intermixed populations of genetically divergent tumor cell populations, containing a minority of cancer stem cells (CSC) and more differentiated cells that make up the tumor bulk. In this review we will use the term of CSC that refers functionally to the tumor cell population with “stemness”, or the capacity to self-renew, generate differentiated cells and sustain tumor proliferation [65]. Intratumoral heterogeneity is thought to contribute to disease progression [66]. In particular, CSCs are thought to be in large part responsible for the inherent radioresistance of a number of primary CNS tumors, including glial tumors and medulloblastoma. Increasing evidence supports the notion that genetic diversity, epigenetics and tumor microenvironment control stemness, which thereby influence patient responses to therapy [67,68].

The genetic diversity of some brain tumors is illustrated by several recent studies that suggest a parallel evolution of genetically distinct subclones within a tumor. Surgical multisampling and molecular analysis of glioblastoma multiforme (GBM) tissues have demonstrated the presence of multiple subclones within a single brain tumor that presumably arise through branching evolution [63]. In another study, single-cell RNA sequencing of GBM tissue revealed not only the presence of different glioblastoma subtypes within a tumor, but also expression of diverse transcriptional programs related to oncogenic signaling, proliferation, complement/immune response, and hypoxia [69]. In addition, single cell analysis of GBM has revealed that different subclones have variable regenerative or stem cell activity [70,71]. The response to genotoxic damage of these different populations may differ and thus differentially impact the response to radiotherapy. CSCs, if not eradicated, can reconstitute a tumor after treatment concludes.

An additional aspect of tumor complexity is provided by the inherent plasticity of tumor cells that allows adaptation to intra- as well as extracellular changes. The recent identification of a core set of neuro-developmental transcription factors (POU3F2, SOX2, SALL2, and OLIG2) that are sufficient to fully reprogram differentiated GBM cells to ‘‘induced’’ tumor promoting cells, puts forth the possibility of bidirectional plasticity through epigenetic reprogramming [72].

Another interesting recent finding that sheds a new light on therapeutic resistance of aggressive brain tumors is the capacity of astrocytoma cells to form tumor microtubes (TM) that create interconnected functional networks [73]. Osswald and colleagues demonstrated that this network of microtubes helps tumor astrocytes to resist cell death by helping dissipating intracellular calcium waves across gap junctions in this network. In addition, it was observed that this network is self-repairing, which also largely protected the connected cells from cell death. Interestingly, down regulation of Cx43, a specific connexin overexpressed in astrocytomas, caused loss of TM. On the other hand, overexpression in oligodendroglioma cells of GAP-43, a protein highly expressed in axonal growth cones, caused the formation of TM in these cells that normally lack them. Thus, Cx43 and GAP-43 could represent new targeting options to increase radiosentivity in astrocytoma.

The role of the microenvironment in sustaining stem cell-like programs in brain tumors has been explored best in the context of the perivascular niche, where nitric oxide released by endothelial cells induces a stem cell state in PDGF-induced gliomas via activation of the Notch pathway [74]. The perinecrotic niche has also been suggested to favor stemness via providing hypoxic conditions that stimulate hypoxia-inducible factor 2a and consequent induction of specific tumor stem cell signature genes [75,76]. Comparing the dispersal of radiation-induced γ−H2AX foci in primary GBM cells grown *in vitro* and within intracerebral xenografts, Jamal and colleagues [77] observed a faster dispersal in vivo, suggesting a greater capacity to repair DNA damage *in vivo* than *in vitro*. Together with data on gene expression that indicated higher expression levels for genes involved in ROS metabolism and antioxidant response in intracerebral xenografts than *in vitro*, these authors suggested that the brain microenvironment protects GBM cells from radiation-induced DNA damage and facilitates its repair.

Several recent comprehensive reviews have described the properties of brain tumor CSCs, the approaches taken to study them and the implications of CSCs for the development of new therapies, as well as the role of the microenvironment in maintenance of glioma stem cells [65,78,79]. Here, we will focus our discussion primarily on those characteristics of CSC that contribute to radioresistance in brain tumors.

## 5. Enrichment of CSC by Ionizing Radiation (IR)

The enhanced resistance of CSC to radiation leads to preferential enrichment of this minor subpopulation following radiation treatment. CD133^+^ brain tumor stem cells were in fact found to be enriched in human glioma xenografts exposed to radiation [80]. These cells, isolated from irradiated mouse cohorts, generated tumors faster than CD133^−^ populations. Rates of post-irradiation apoptosis were lower in CD133^+^ cells compared to non-stem populations [80]. Another mechanism of CSC enrichment by radiation has been hypothesized to be due to conversion of differentiated tumor cells to stem-like cells, a process which may be stimulated by environmental stressors such as hypoxia and radiotherapy [81]. In support of this, Dahan and colleagues observed an increase in neurosphere formation after exposure of differentiated glioblastoma cells to a sublethal dose of ionizing radiation (IR) [82]. However, an alternative explanation of these findings could be that IR promoted a shift from asymmetric to symmetric division of stem cells, as has been proposed to occur after temozolomide treatment [83]. Although convincing proof of IR-induced dedifferentiation as a mechanism of CSC enrichment is still lacking, dissection of the molecular underpinnings of this phenomenon could yield novel targets for radiosensitization.

## 6. DNA Damage Response

The main mechanism through which radiotherapy kills tumor cells is by causing single and double stranded DNA breaks, which, if not repaired, induce cell death. Actively dividing cells are more susceptible to damage by radiation, with cells being most sensitive while in G2-M, less so in G1 and least radiosensitive in late S phase [84]. An important trait of CSCs is their slow rate of cell division found in certain CSC populations in several tumor types [85]. Slow-cycling CSC have been found also in glioblastoma where they have been implicated in resistance to genotoxic damage caused by chemotherapy [85,86]. It is, thus, conceivable that this property of this type of cells may also contribute to radioresistance in brain tumors.

In response to double-stranded DNA damage, cells mount a complex DNA damage response [87]. This is initiated by the MRN (MRE11-RAD50-NBS1) complex which senses double strand breaks and activates the serine/threonine protein kinases ataxia telangiectasia mutated (ATM) and ataxia telangiectasia and Rad3-related (ATR). These proteins then phosphorylate and activate the checkpoint kinases Chk1 and Chk2, leading to cell cycle arrest, as well as a set of proteins that are involved in repairing the DNA damage [88]. Glioblastoma CSCs are endowed with an enhanced response to DNA damage, which contributes to their radioresistance [80]. GBM-derived CSCs have enhanced basal activity of DNA damage response targets that include ATM, ATR, Chk1 and Chk2, which contribute to a stronger G2/M checkpoint activation and subsequent DNA repair [89]. Bao and colleagues [80] showed that CD133^+^ GBM progenitor cells possess a robust response to DNA damage, which involves detection and repair mechanisms that render them significantly more radioresistant than their differentiated counterparts. ATM kinase inhibition was shown to lead to enhanced radiosensitization in glioblastoma CSCs [90]. Likewise, targeting Chk1 and Chk2 with the checkpoint kinase inhibitor debromohymenialdisine was able to overcome radioresistance in CD133^+^ cells in both *in vitro* and *in vivo* [80].

Tumor cells that are genetically deficient in at least one DNA repair pathway can be sensitized to radiation by targeting other redundant pathways [91]. This phenomenon is termed synthetic lethality [92,93]. A key target to generate synthetic lethality is the enzyme poly [adenosine diphosphate (ADP)] ribose polymerase (PARP). As a critical factor in the base excision repair (BER) pathway, PARP is involved in the repair of single strand DNA breaks (SSB), part of the sublethal damage generated after exposure to IR [94]. PARP recognizes the free ends of DNA and recruits other factors necessary for repair to occur properly [95]. PARP inhibitors including ABT-888 (veliparib) [96,97,98], AZD-2281 and E7016 have been shown to cause *in vitro* and *in vivo* radiosensitization of glioma and are currently being tested in phase I and II trials to assess treatment response in CNS tumors [99].

Although elements of either the DNA damage checkpoint response or DNA repair proteins are in principle good targets for radiosensitization, simultaneous targeting of both pathways has been shown to be more effective. Inhibition of ATM that affects both cell cycle checkpoint regulation and DNA repair, achieved greater radiosensitization compared to inhibition of CHK1, ATR or PARP alone [100]. Thus, molecules that signal upstream of these responses may represent good therapeutic targets. One such example is L1CAM (CD171), a CSC marker and cell surface adhesion protein that causes phosphorylation and activation of ATM, Rad17, Chk1 and Chk2 after IR, enhancing the DNA damage response. L1CAM also induces expression of NBS1, a member of the MRN complex that controls cell cycle checkpoint as well as DNA repair after DNA damage. L1CAM down regulation sensitized glioblastoma stem cells to radiation [101]. Thus, L1CAM signaling could provide a novel target to overcome radiation resistance.

Another signaling element that confers radioresistance by activating CHK2 in response to IR is the stress activated protein MRK, also known as ZAK [102]. Although its involvement in CSC radioresistance has not yet been explored, MRK has been shown to be activated by IR downstream of ATM and NBS1 and to be necessary for complete activation of CHK2 and consequent cell cycle arrest [103,104]. Interference with MRK expression by its down regulation, or inhibition of its activity by a specific small molecule, in medulloblastoma cells leads to failure to arrest cell cycle division and enhanced IR-induced cell death (RR, [105]).

In response to IR, p53 is implicated as a major regulator of radiation responsive genes that are believed to contribute significantly to radioresistance. Adenovirus-mediated expression of p53 in glioblastoma cells has been shown to enhance radiosensitivity [106]. *In vivo* expression analysis using the Olig2-TRAP transgenic system to examine transcription and translation simultaneously on a genome-wide scale, have identified the p53 target gene cluster to be among the most robust transcriptional and translational response clusters noted in proneural GBM in response to IR. These findings provide support to the notion that p53 plays a key role in modulating the radioresistant phenotype in glioblastoma by driving transcription of apoptotic gene expression programs and that of genes that are involved in stress responses, including redox imbalances [107]. Thus, p53 mutations or loss of wt p53 can cause radioresistance. As p53 is essential for the G1 checkpoint cell cycle arrest, tumor cells that have lost its function rely on the S and G2/M checkpoints for stopping cell division after IR. Therefore, an approach that has been explored to radiosensitize p53 mutant cell is to interfere with elements that are responsible for the S and G2/M checkpoints. The use of Chk1 and PARP1 inhibitors have been shown to radiosensitize p53 mutant pancreatic cells and the ATM kinase inhibitor KU-60019 preferentially sensitized p53-mutant glioma *in vivo* [108].

In addition to the DNA damage checkpoint proteins, an avenue that has been explored to prevent cell cycle arrest after DNA damage has been the inactivation of regulators of the cyclin-CDK complex that controls cell cycle progression. One such element is WEE1, a potent inhibitory kinase that directly regulates cyclin-dependent kinase 1-mediated cell cycle progression through the G2/M phase into mitosis. In response to DNA damage, cell division is halted by WEE1 to allow time for effective DNA repair [109,110]. An *in silico* analysis found that WEE1 is highly expressed in high grade glioma compared to normal brain tissue and siRNA-mediated depletion of WEE1 led to abrogation of G2 checkpoint-mediated cell cycle arrest and increased cell death [111]. Use of WEE1-selective inhibitors such as AZD1775 (formerly MK-1775) has been shown to radiosensitize glioblastoma and diffuse intrinsic pontine glioma cells and phase 1 clinical trials are now testing this in the clinic [109,112,113,114].

Radiation causes DNA damage by indirect energy transfer that produces chemically reactive free radicals, including ROS. In addition to an effect on DNA, radiation may induce cell death via damage of critical cellular structures like lipids and proteins [115]. Interestingly, low levels of ROS have been found in CSCs and are attributed not only to lower production, but also to increased expression of ROS scavenging systems, such as glutathione [116]. Thus, ROS scavenging systems might represent novel targets to enhance radiation response.

## 7. Signaling Pathways

Intrinsic factors include survival signaling pathways that are deregulated through alterations in growth factors or their receptors, up-regulation of pro-survival proteins, such as Bcl2 family members, and changes in the DNA damage response and in metabolism. Although these pathways are usually studied in isolation and in large part are considered to be independent, they are in fact interrelated. Growth factor signaling for instance can lead to deregulated anti-apoptotic signals, as well as improved DNA damage response [117,118]. The potential interactions among the different signaling pathways may in fact contribute to escaping targeted therapeutic interventions that are aimed at individual pathways.

Intracellular signaling is further modulated by signals contributed by the microenvironment and conditions such as angiogenesis and hypoxia. The contribution of the microenvironment to maintenance of the stem cell phenotype has been extensively discussed in recent reviews [78,119]. In this capacity the microenvironment can enhance radioresistance of brain tumors. One of the signaling pathways implicated in radioresistance *in vivo* is the one generated by the pleiotropic cytokine transforming growth factor-β (TGF-β). Its inhibition by LY364947 or by LY109761, two small molecule inhibitors of TGF-β type I receptor kinase, in combination with radiotherapy, has been shown to interfere with DNA damage repair, thereby improving survival in preclinical studies [120,121]. Among the various cell types that comprise the microenvironment, microglia, the brain resident macrophages, have also been shown to confer radioresistance to tumor cells. Inhibition of microglia activation by semapimod was shown to sensitize glioblastoma tumors to ionizing radiation [122].

Another important problem recognized for decades in radiobiological research, is that of intratumoral hypoxia and its role in radioresistance [123,124,125]. Strategies including the use of hyperbaric oxygen to improve oxygen supply to tumors or drugs that specifically target and radiosensitize hypoxic tumor cells have been exhaustively pursued [123,126,127,128]. However, it is evident from Jens Overgaard’s 2007 meta-analysis of 82 hypoxic radiosensitization clinical trials that the majority of trials evaluating CNS tumors treated with nitroimidazole-derived compounds as potentially useful radiosensitizers failed to show any clinical benefit [129]. Other approaches have tried to specifically target hypoxic cells in malignant gliomas with systemic therapies such as mitomycin or tirapazamine that are more toxic to these hypoxic tumor cells than normoxic cells, but without apparent clinical benefit [130,131]. Nevertheless, new data elucidating molecular networks responsible for hypoxia-mediated radioresistance of CNS tumors are encouraging. They include the discovery of potentially drugable targets upstream and downstream of hypoxia inducible factor-regulated pathways [76,78,132,133].

**Figure 1:**
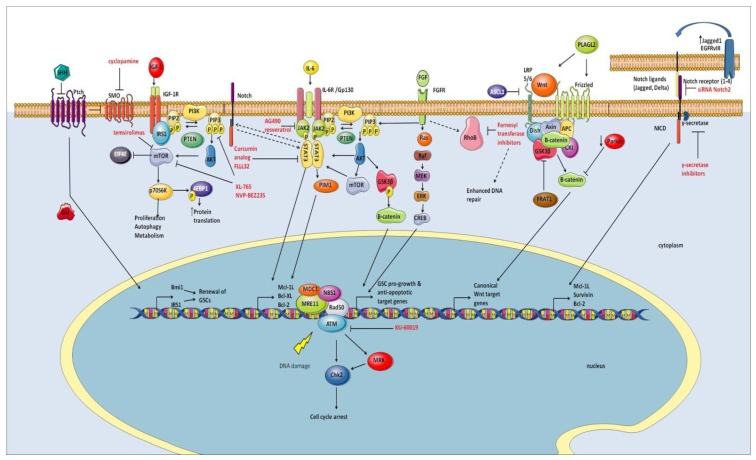
Schematic representation of important signaling pathways involved in radiosensitization. See text for detailed description.

Although the mechanisms through which the microenvironment supports radioresistance in brain tumors largely remain to be elucidated, its importance is widely recognized and it continues to be the subject of active studies. In the following chapters, we will focus our discussion on signaling elements that directly contribute to radioresistance of cancer stem cells (see Figure 1 for a schematic of the pathways discussed herein).

### 7.1. Notch

One of the important signaling elements associated with maintenance of stemness is Notch and its downstream pathway. This evolutionarily highly conserved pathway is initiated by binding of the Notch receptor to its ligands on adjacent cells. This leads to Notch intracellular domain (NICD) release after cleavage by γ secretase-like protease. NICD subsequently translocates into the nucleus and binds to specific response elements to transactivate target genes [134]. Notch plays complex roles during normal vertebrate neural development. It promotes neural stem cell survival and self-renewal [135] and inhibits differentiation of neural progenitor cells into neurons [136]. However, Notch signaling also promotes terminal differentiation of astrocytes from glial progenitor cells, while inhibiting the differentiation of oligodendrocytes [136].

Early studies had shown that Notch blockade using a γ secretase inhibitor in medulloblastoma cells depleted CD133^+^ cells, and treated cells showed diminished soft agar colony formation and tumor growth, indicating that Notch signaling is necessary for the maintenance of medulloblastoma stem cells [137]. Over the past decade, accumulating evidence also has shown a critical role for Notch signaling in the maintenance of glioma stem cells [78,138]. In addition, analysis of Notch signaling elements revealed increased expression of Notch ligands (DLL1, DLL3), Notch receptors (NOTCH1, NOTCH2) and target genes (HES1, HES5, HEY1) in multiple primary tumors of the CNS including astrocytoma, ependymoblastoma, oligodendroglioma, meningioma and GBM compared to normal tissue matched controls [139]. Interestingly, proteomic analysis of a set of 27 surgical glioblastoma samples revealed that activation of Notch signaling is highly correlated with EGFR activation [140], a hallmark of the classical subtype of GBM tumors [141]. In line with this, Jagged1 is the most abundant Notch ligand in GBM and its transcription is stimulated by EGFR variant III (EGFRvIII) [142]. We note, however, that expression analysis of The Cancer Genome Atlas glioblastoma dataset also identified high Notch activity in a subset of proneural-type GBM, correlating with high responsiveness to γ secretase inhibitors [143], a discrepancy that may be due to the lack of correspondence between protein levels and mRNA levels.

Importantly, the Notch pathway plays a critical role in the radioresistance of glioblastoma stem cells. Interference with Notch signaling with a γ secretase inhibitor has been shown to impair clonogenic survival of CD133^+^ stem cells, but not of CD133^−^ cells [144]. Knockdown of Notch1 or Notch2 also increased the radiosensitivity of glioma stem cells. Notch-mediated radioprotection is thought to be mediated in part by activation of the Akt and altering the balance between the truncated apoptotic isoform of Mcl-1 (Mcl-1_S_) and Mcl-1_L_ toward increased expression of the latter pro-survival protein [144]. The efficacy of γ-secretase inhibitors in the clinic remains to be evaluated.

### 7.2. Wnt/β-Catenin Pathway

Another pathway implicated in the maintenance of stem cells is the canonical Wnt/β-catenin pathway, which is an important regulator of embryonic development as well as of adult stem cells and it has been found to be altered in cancer [145,146]. Upon binding of Wnt ligands to receptors of the Frizzled family and low density lipoprotein receptor-related protein/α2-macroglobulin family (LRP), β-catenin is stabilized and translocates to the nucleus, where it activates the T-cell factor/lymphoid enhancer factor (TCF/LEF) to transcribe Wnt target genes [146].

The Wnt/β-catenin pathway is involved in radioresistance of GBM in part through maintaining the stem cell phenotype. This pathway was found to be activated within the stem cell population in GBM orthotopic mouse models that had been treated with radiation. Inhibition of the Wnt pathway led to decreased survival and clonogenicity of GBM cells, as well as reduction of the glioma stem cell subpopulation [147]. Another potent oncogene, PLAGL2 (pleomorphic adenoma gene like 2), a putative zinc finger transcription factor discovered on 20q11.21, a commonly amplified genomic region in GBM, was identified as a Wnt-pathway activator that leads to suppression of both neural stem cell and glioma initiating-cell differentiation. Thus, PLAGL2 acts as a powerful driver of stem cell induction, maintenance and proliferation downstream of Wnt activation [148].

Within glioma, Wnt and its cofactors are highly deregulated, contributing to increased tumorigenicity. As an example, the Wnt family of embryonic lineage specifying genes, SFP1 and SFP2 (soluble Frizzled-related proteins) are often over-activated in glioma. It appears that these proteins both promote glioma tumor growth and survival via enhanced clonogenicity and survival in the absence of growth factors [149].

Control over Wnt signaling in glioma is often mediated in an epigenetic fashion. For example, the gene promoters of Wnt pathway inhibitor genes, including soluble frizzled related proteins, Dickkopf and naked genes, were found to be heavily methylated leading to epigenetic silencing [150]. In another study by Rheinbay and colleagues [151], an epigenomic analysis comparing chromatin landscapes of GBM CSCs to human astrocytes, identified Achaete-Scute Complex-like 1 (ASCL1) as an important activator of Wnt signaling from a list of aberrantly expressed genes normally kept inactive by Polycomb receptors. ASCL1 removes the negative regulation of Wnt, normally imposed by Dickkopf Wnt signaling pathway inhibitor 1 (DKK1), a process that was shown to be necessary for glioma CSC tumorigenicity.

Other means of Wnt pathway deregulation in glioma involve FRAT1 (frequently rearranged in advanced T cell lymphoma) that works to inhibit β-catenin phosphorylation by GSK. FRAT1, thus, acts as a potent oncogene in the induction of Wnt signaling and positively correlates with increasing grade of glioma [152,153]. Wnt-mediated tumorigenicity appears indeed to be β-catenin-dependent, as shown by Wang and colleagues [154]. They showed that alteration of β-catenin activity via suppression of pygopus 2 gene expression, a regulator of β-catenin/Tcf-mediated transcription downstream of Wnt, leads to reduced cyclin D1 expression, thereby increasing cell cycle arrest in G1 and reducing cell proliferation. Not surprisingly, siRNA targeting of β-catenin, which is elevated in immunohistochemical analysis of glioma specimens, also arrested cells in G1 and caused decreased activation of c-Myc, c-jun and cyclin D1, while also inducing apoptotic cell death in glioma cells. In glioma, key regulators of the Wnt/β-catenin pathway such as the ubiquitin-E3 ligase, Parkin (PARK2) are often under-expressed or deleted. PARK2 is an ubiquitin-dependent negative regulator of β-catenin and restoration of its function leads to the attenuation of glioma proliferation [155].

In contrast to GBM, Wnt pathway activation in medulloblastoma is associated with favorable prognosis [156] and its exogenous expression in medulloblastoma cells contributes to increased radiosensitivity [157].

### 7.3. SHH/Gli Pathway

A third signaling pathway implicated as a central mediator in the pathogenesis of glioma and glioma stem cells (GSCs) is the Sonic Hedgehog (SHH). SHH binds to its transmembrane receptor patched (PTCH) to release Smoothened (SMO) from PTCH mediated-inhibition, which in turn initiates activation and nuclear translocation of Kruppel zinc finger transcription factor Gli1 [158,159,160]. Target genes vital to “stemness” and self-renewal properties of GCSs are transactivated by the Gli family after SHH activation [161]. Among these targets, insulin receptor substrate 1 (IRS1) appears to play a role in IGF-mediated self- renewal of GSCs [162].

Up regulation of the Hedgehog signaling has been shown in glioblastoma and medulloblastoma, and to a lesser extent, in neuroblastoma [163]. In medulloblastoma, SHH/Gli has been shown to drive expression of stem cell modulator Bmi1, a key transcriptional polycomb repressor, found to be overexpressed in medulloblastoma and in CD133^+^ tumor initiating progenitors [164,165,166,167]. Bmi1 is known to be vital to the sustained self-renewal and stemness properties of human GSCs [168]. Bmi1 has also been implicated in the DNA damage response pathway, both through regulation of mitochondrial function with regard to the cellular redox state [169] and through modulation of DNA double strand break repair efficacy by enabling targeted ubiquitination and degradation of γ-H2AX. Loss of Bmi1 causes significant radiosensitization [170].

Sonic Hedgehog pathway blockade, established through treatment of medulloblastoma with cyclopamine, resulted in neuronal differentiation and decreased stemness characteristics *in vitro* [171]. In glioma, cyclopamine was shown to inhibit growth in cell lines expressing high levels of Gli1. Cyclopamine treatment also diminished clonogenicity, arguing for the dependence of stemness on the SHH/Gli1 pathway [172].

Because of the prominent role of the hedgehog pathway in cancer, and despite limited success so far, several antagonists of this signaling cascade are still being tested in the clinic [173,174].

In addition to regulators of embryonic development like the ones described in the above sections, a number of growth factors and their receptors have been strongly implicated in stem cells maintenance and in radioresistance and are discussed below.

### 7.4. FGF-2

Fibroblast growth factor-2 belongs to the FGF superfamily that comprises 22 different genes. FGF-2 signals primarily through the fibroblast growth factor receptors (FGFRs) FGFR-1 and FGFR-2, which lead to activation of the Ras-MAPK and the PI3K-Akt pathways [175]. The derived signals promote cellular growth and antagonize apoptosis. In glioma, FGF-2 contributes to maintenance of glioma stem cells and it is used in the media that support their growth [176]. Growth factor withdrawal resulted in differentiation of glioma stem cells, not observed in the presence of growth factor [177]. Lathia and colleagues [178] also observed that symmetrical division of glioma stem cells *in vitro* depends on FGF-2 and that its removal favored differentiation.

Several studies have implicated fibroblast growth factor signaling in radioresistance. Early studies revealed a role for FGF in the protection of endothelial cells from apoptosis [179], a process thought to be dependent on protein kinase C-mediated signaling downstream of FGF [180]. Later, FGF-2-stimulated radioresistance mechanisms were further elucidated by linking the small GTPase farnesylated RhoB as a downstream mediator of radioprotection downstream of FGF-2 [181]. Inhibition of RhoB function by a farnesyltransferase inhibitor was found to sensitize glioma cells to ionizing radiation [182]. In response to IR, RhoB, along with αvβ3 and 5 via integrin-linked kinase signals protected against radiation-induced mitotic cell death [183,184]. In addition, RhoB promoted γ−H2AX dephosphorylation and DNA double strand breaks repair after IR exposure [185]. The FGF pathway is an attractive target for development of radiosensitization agents to facilitate effective treatment of glioblastoma as its overexpression in patient GBM tumor samples correlates with inferior survival. In fact, FGFR-1 has been identified as an independent risk factor for poorer prognosis with decreased time to progression in patients whose tumors express this receptor [186]. A small molecule designed to block FGFR has been shown to increase progression free survival in mice with GBM in response to radiation, further confirming the importance of the FGF pathway in radioresistance of brain tumors [187].

### 7.5. EGFR

During normal CNS development, the receptor tyrosine kinase, epidermal growth factor receptor (EGFR) plays a major role in facilitating glial progenitor survival throughout gliogenesis. Transgenic overexpression of the receptor suggested a role for EGFR in driving glial proliferation and in promoting neural stem cell survival [188,189].

EGFR is frequently found to be amplified in highly proliferative tumors such as glioblastoma, where it occurs in about 50% of cases [190]. Constitutive activation of EGFR signaling in glioblastoma occurs also through deletion of exons 2–7 in the EGFR mRNA that leads to expression of the EGFRvIII variant [191,192]. This constitutively active mutant receptor lacks part of the extracellular domain, which renders it unable to bind ligands. It is found in approximately 40% of grade IV tumors with EGFR amplification [192]. EGFR has been shown to support tumorigenicity primarily through signaling toward downstream targets including RAS/RAF/MAPK and PI3-K/AKT pathways, which in turn leads to increased proliferation, angiogenesis, migration and invasion of glioma cells [189,193].

There is strong evidence that antagonism of EGFR leads to radiosensitization in high grade glioma. Early preclinical work using EGFR tyrosine kinase inhibitors, such as AG1478, or dominant negative constructs including EFGR-CD533, have all caused significant radiosensitization with dose enhancement ratios approaching 1.85 [189,194,195,196,197].

Several clinical trials have tested various small molecule and antibody-based EGFR tyrosine kinase inhibitors (TKIs), while vaccine-based and siRNA mediated EGFR targeting trials are now underway. To take advantage of the radiosensitizing effects of EGFR attenuation seen in pre-clinical models, phase I and II clinical trials designed to test the efficacy of an anti-EGFR monoclonal antibody coupled to radioactive iodine (^125^I-mAb 425) have been conducted. Results from a large phase II trial testing this strategy were encouraging with a median survival of 20.2 months when administered with TMZ [198]. However, the EGFR TKIs gefitinib and erlotinib and the monoclonal antibodies cetuximab, panitumamab and nimotuzumab, given adjuvantly, have failed to show efficacy in treating high-grade glioma in clinical trials up to this point. This is due likely in large part to issues with blood brain barrier permeability to the targeted agents and acquired resistance to EGFR inhibition [199]. In particular, inherent resistance to these therapies is thought to be secondary to the glioma stem cell compartment’s ability to activate alternative pathways to bypass EGFR upstream signals [200].

### 7.6. IGF

The role of insulin like growth factor signaling in radioresistance has been well established in many extracranial tumor types. As alluded to in the sonic hedghog section, IGF signaling is necessary for GSCs to maintain proliferative and self-renewal capacities downstream of SHH/Gli signaling. In this context, the Gli transcription factor transactivates insulin receptor substrate I to drive MAPK activation and to facilitate downstream GSC functions that include proliferation, self-renewal, invasion and angiogenesis [162].

IGF-1 receptor signaling is important in glioma stem cell resistance to ionizing radiation. Experiments done with murine glioma stem cells, exposed to fractionated radiation, showed upregulation of IGF-1 receptor and increased IGF-1 secretion, leading to enhanced self-renewal of glioma stem cell populations along with Akt activation. Furthermore, treatment with an IGF-1 receptor inhibitor resulted in significant attenuation of AKT-mediated survival and subsequent radiosensitization [201]. Thus, IGF-1 receptor inhibitors may represent a new class of radiosensitizers for gliomas.

### 7.7. cMet

cMet is recognized as a neurotrophic protein, vital to the normal CNS development and to the propagation of malignant gliomas [202]. cMet expression correlates with increased tumor grade [202,203]. Activation of its tyrosine kinase domain by binding of hepatocyte growth factor/ scatter factor (HGF/SF) in glioblastoma cells is thought to stimulate a more invasive phenotype and to promote tumor angiogenesis [204]. cMet signaling reduces apoptosis in response to DNA damage triggered by ionizing radiation, via phosphorylation of anti-apoptotic AKT signal transduction pathways [205]. Synergism to induce greater GBM tumor cell killing has been shown with the combination of hypofractionated ionizing radiation and SF/HGF/cMet blockade, via U1snRNA/ribozymes. This treatment also led to decreased blood vessel formation within tumors [206,207].

Many of the signaling elements discussed above utilize common intracellular signals. Among them, the PI3K/Akt/mTOR pathways have a central role in cell survival and in the response to radiation.

### 7.8. PI3K/Akt Pathway

As mentioned in some of the previous sections, Akt is a key pro-survival signaling kinase downstream of many growth factors and of oncogenic receptor tyrosine kinases [208,209]. Hyperactivation of Akt signaling has been correlated with worse progression-free survival and overall survival in GBM patients [210,211,212]. Aberrant activation of Akt in GBM can occur as a result of loss of PTEN suppressive function, which leads to unchecked proliferation through an array of pro-growth and anti-apoptotic mechanisms [213,214]. In addition, the PI3K/Akt pathway can be abnormally activated as a consequence of epidermal growth factor receptor (EGFR) gene amplification and rearrangement, as well as CTMP promoter hypermethylation [215,216]. Importantly, Akt is required for the maintenance of a stem-like state in glioma [217] and Akt inhibition increases their rate of apoptosis and decreases the ability to form neurospheres [218,219]. Aberrant Akt activation has also been observed in medulloblastoma [220].

Inhibition of Akt signaling in glioma cells has radiosensitizing effects [221]. Radiosensitization in response to either Akt or PI3K inhibitors, or introduction of functional PTEN, has been demonstrated in U87 glioma cells [222]. Similar radiosensitization was observed in primary patient-derived glioblastoma cells, which were found to be sensitive to a low dose of an Akt inhibitor [223]. Thus, Akt seems to be an attractive target for GBM therapy.

### 7.9. mTOR

Acting downstream of Akt, the mTOR serine/threonine protein kinase is a master regulator of cell growth, proliferation, metabolism and autophagy, a cellular recycling and degradation system [224]. Signals propagated through mTOR originate from insulin growth factor receptor to affect amino acid metabolism and transport. In this way, mTOR acts in response to the metabolic and nutritional state of the cell and surrounding microenvironment. In the presence of ample supplies of extrinsic amino acids, mTOR kinase activation increases anabolic cellular functions including translation through S6K1-dependent phosphorylation and subsequent induction of eukaryotic initiation factor 4E (4EBP1) [225].

The two major mTOR complexes, mTORC1 and mTORC2, play critical roles in the oncogenesis of primary CNS neoplasms and contribute to radioresistance [226]. In pediatric gliomas, 80% of the high-grade and 50% of low-grade gliomas have enhanced activation of two of the major downstream targets of mTORC1, S6 and 4EBP1, which correlates with decrease in progression-free survival independently of tumor grade [227].

The mTOR pathway has been shown to be highly activated in medulloblastoma stem cells within the perivascular niche after radiation and Akt inhibition in this model sensitized cells within this niche to radiation-induced apoptosis [228]. A radioresistant phenotype has also been observed in glioma cells through an mTOR-dependent autophagy [229]. Inhibition of autophagy in glioblastoma cells, using a dual pI3K/ mTOR inhibitor (NVP-BEZ235), led to increased radiosensitization in both *in vitro* and *in vivo* glioma models [230,231,232]. Work from Lomonaco and colleagues shows autophagy to be increased in IR-resistant clones derived from treated glioblastoma cancer stem cells [233]. However, the notion that autophagy causes greater radioresistance is still controversial, as Nam and colleagues [234] reported that mTOR inhibition-dependent enhancement of autophagy in glioma cells, after exposure to ionizing radiation, leads to reduced tumor size, which was caused by premature cellular senescence.

The hypoxic microenvironment within GBM leads to resistance to ionizing radiation. This has been found to be in part secondary to mTOR-dependent inhibition of translation, via modulation of eukaryotic initiation factor for E (EIF4E), the key factor involved in cap-dependent protein translation. Bypassing mTOR repression, via overexpression of EIF4E, leads to enhanced radiosensitivity and selective loss of the hypoxic cell population [235].

Despite promising pre-clinical data, recent phase I and II clinical trials using mTOR pathway inhibitors, including temsirolimus monotherapy, have failed to demonstrate improved overall survival in recurrent glioma patients, although various radiographic responses were observed [236]. Two phase I trials combining temozolomide with mTOR inhibitors and concurrent radiotherapy have been conducted, showing the treatment to be well tolerated with decreases in tumor metabolism noted on PETCT-based imaging [237,238]. These disappointing results may be explained in part by pre-clinical and phase I data that have suggested potential loss of negative feedback regulation over Akt, derived from the use of mTOR inhibitors alone or combined with standard chemoradiotherapy in the treatment of high grade glioma. This observation provides support for the employment of dual PI3K/mTOR inhibitors [239]. Indeed, pre-clinical data generated using dual inhibitors have justified a currently ongoing phase I clinical trial combining dual PI3K/mTOR inhibitor XL765 with concurrent chemoradiation [239,240,241]. Further support for combined inhibition of multiple interconnected pathways is provided by work done in GBM with blockade of endogenous EGFR activity concomitantly with mTOR inhibition, leading to enhanced radiosensitivity mediated through pro-death autophagy [242].

Finally, downstream of intracellular signaling elements, transcription factors are responsible for regulation of gene expression and for implementing new cell programs. Here we review the role of two crucial transcription factors in establishing radioresistance.

### 7.10. NF-κB

Classically, nuclear factor-kappaB (NF-κB) functions as a transcription factor kept under tight cytoplasmic regulation by the kappa B inhibitor protein, IκB. Upon ligand binding of a cell surface receptor such as TNF-R or Toll-like receptor, IκB is targeted for proteosomal degradation via IKK complex-mediated phosphorylation, thus releasing NF-κB to translocate into the nucleus and transactivate downstream target genes [243].

NF-κB activation was recently shown to contribute via MLK4 to the development of intratumoral heterogeneity and enhanced radioresistance of glioblastoma by inducing a transition from a proneural to mesenchymal transcriptional GBM subtype [244,245]. Furthermore, radiation-dependent TNFα activation is able to enhance NF-κB-mediated radioresistance, while inhibition of NF-κB signaling promotes TNFα-mediated apoptosis in response to the radiomimetic neocarzinostatin via p53-dependent TIGAR activation [246]. In response to radiation, NF-κB is thought to promote resistance to radiation-induced cell death downstream of PI3K/AKT via the promotion of anti-apoptotic genes such as Bcl-2 and Bcl-XL [247]. For example, in response to ionizing radiation induced- DNA-damage, NF-κB is activated to drive the expression of IL-6, IL-8 and Bcl-XL and promotes GBM proliferation via a miR-181b-mediated positive feedback loop, which enhances NF-κB activity downstream of IR [248].

Early *in vitro* attempts at targeting NF-κB activity in GBM have been successful in decreasing tumor cell proliferation. One approach targeted tumors cells with poly (DL-lactic-co-glycolic acid) (PLGA) microparticle-containing NF-κB decoy oligonucleotides possessing DNA-binding sites specific for the wild-type NF-κB DNA-binding domain. Release of the decoy oligonucleotides within the cell acts to bind up free NF-κB, thus effectively inhibiting transactivation of downstream target genes, leading to inhibition of GBM cell growth and proliferation [249,250]. Other selective NF-κB inhibitors include dehydroxymethylepoxyquinomicin that in combination with temozolomide (TMZ) was shown to synergistically sensitize cells to radiation [251]. Additionally, a phase I/II clinical trial utilizing the NF-κB inhibitor, sulfasalazine was recently closed after an interim analysis failed to demonstrate efficacy when used as second-line monotherapy in the treatment of patients with recurrent GBM [252,253]. However, trials looking at a combined approach with radiation have not yet been carried out.

### 7.11. STAT3

STAT3 functions at the confluence of several important molecular pathways involved in radioresistance of brain tumors, including Wnt, Notch, Akt and the Bcl2, Bcl-xL and Mcl-1 survival genes [254]. STAT3 is a transcription factor that acts downstream of the gp130 receptor upon binding to IL-6. Receptor activation leads to STAT3 phosphorylation, dimerization and translocation into the nucleus, where it transactivates pro-survival genes such as c-Myc, Bcl-xL, MCl-1, and survivin to promote cell proliferation [255,256,257]. STAT3 can also be activated downstream of other receptors, including EGFR, as well as by receptor-associated kinases such as JAK2 and Src [258,259,260]. STAT3 has been shown to be constitutively activated in gliomas and medulloblastomas [261,262,263,264].

STAT3 is also implicated as a key driver in the proneural to mesenchymal shift seen in GBM after exposure to RT, which contributes to a radioresistant phenotype [107]. Phosphorylation of serine 727 is important for STAT3-mediated resistance to ionizing radiation in glioma and, thus, disrupting phosphorylation at this site could provide a potential target for radiosensitization [265]. Novel inhibitors targeting STAT3 have been rationally designed, such as FLLL32. This molecule, modified from curcumin, leads to significant decreases in S727-STAT3 phosphorylation in established glioblastoma cell lines [266]. The Jack2 inhibitor, AG490, as well as resveratrol, were shown to inhibit not only the stem-like properties of GBM cells, but also to reduce their radioresistance *in vitro* and *in vivo* [267]. Thus, both molecules could have therapeutic benefits in the clinic.

## 8. Conclusions

Our understanding of the molecular mechanisms that promote radioresistance has revealed a number of potential targets, inhibitors of which could be developed as radiosensitizers. A number of these inhibitors have been tested in the clinic as monotherapy and shown very disappointing results. As it is expected that their benefit as radiosensitizers depends on their concurrent use with radiation, new clinical trials that evaluate these agents in conjunction with radiotherapy will be necessary. The understanding that cancer stem cells represent a source of therapy resistance and failure has suggested that targeting this cell population within the tumor will provide increased sensitization to radiotherapy as well. Thus, together with optimized radiation delivery modalities, advances in identification of radioresistance targets and development of their inhibitors may provide better future therapeutic outcomes for brain tumors. Therapeutic success of small molecule radiosensitizers will depend also on their ability to cross the blood brain barrier, which represents an outstanding challenge in brain tumor treatments. Thus, development of novel delivery modalities that overcome this obstacle will be crucial to improving therapies for brain tumors. As several chemotherapies work by causing DNA damage, it is expected that novel radiosensitizers may be effective also to overcome chemoresistance.
